# Supplementary material for: Influence of Silver Nanoparticles on the Growth of Ascitic and Solid Ehrlich Adenocarcinoma: Focus on Copper Metabolism
Source: Pharmaceutics. 2023 Mar 29;15(4):1099. doi: 10.3390/pharmaceutics15041099 (PMC10145613; doi:10.3390/pharmaceutics15041099)
Supplement: Supplementary file 1 [file pharmaceutics-15-01099-s001.zip › pharmaceutics-2184526-supplementary.pdf]

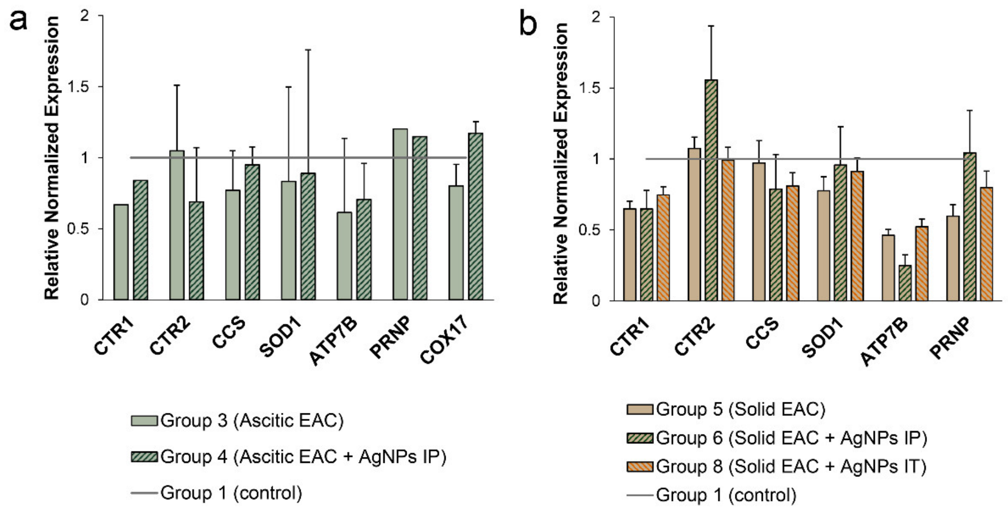

Figure S1. Expression of genes responsible for copper status of blood serum in the liver of mice with (**a**) ascitic EAC treated with AgNPs IP or (**b**) solid EAC treated with AgNPs IP or IT. Group numbers are the same as in Table 1.
